# An approach to sociotechnical transparency of social media algorithms using agent-based modelling

**DOI:** 10.1007/s43681-024-00527-1

**Published:** 2024-07-29

**Authors:** Anna Gausen, Ce Guo, Wayne Luk

**Affiliations:** Imperial College London, London, UK

**Keywords:** Recommendation algorithms, Transparency, Social media, Agent-based modelling

## Abstract

The recommendation algorithms on social media platforms are hugely impactful, they shape information flow and human connection on an unprecedented scale. Despite growing criticism of the social impact of these algorithms, they are still opaque and transparency is an ongoing challenge. This paper has three contributions: (1) We introduce the concept of *sociotechnical transparency*. This can be defined as transparency approaches that consider both the technical system, and how it interacts with users and the environment in which it is deployed. We propose sociotechnical approaches will improve the understanding of social media algorithms for policy-makers and the public. (2) We present an approach to sociotechnical transparency using agent-based modelling, which overcomes a number of challenges with existing approaches. This is a novel application of agent-based modelling to provide transparency into how the recommendation algorithm prioritises different curation signals for a topic. (3) This agent-based model has a novel implementation of a multi-objective recommendation algorithm that is calibrated and empirically validated with data collected from X, previously Twitter. We show that agent-based modelling can provide useful insights into how the recommendation algorithm prioritises different curation signals. We can begin to explore whether the priorities of the recommendation algorithm align with what platforms say it is doing and whether they align with what the public want.

## Introduction

Social media platforms have had a transformative impact on society. As a technological, cultural, and social advancement, they stand out for transforming users, and therefore the public, into “active participants” instead of “passive recipients” of online information [1]. The scale of users and information on these platforms is unprecedented. Originally, many platforms sorted the posts on users’ newsfeeds in reverse chronological order. However, as the scale of information grew, this approach became insufficient and platforms developed recommendation algorithms to curate the content that users see based on predicted engagement.

There has been growing criticism of these algorithms, and social media more widely, for worsening mental health [2], amplifying hate speech [3], spreading misinformation [4], and other negative outcomes [5]. However, these algorithms are opaque meaning that both policy-makers and the public are unclear on the extent to which they are to blame for societal issues. The current approach of self-regulation solidifies the “information asymmetries” [6] between the platforms and the public. There is a need to develop tools for external transparency [7] as a first step towards improved understanding of the algorithms and regulation of the platforms. Transparency can have many meanings [8] but here it refers to the ability to understand how an AI system reaches its decisions.

This paper presents a novel approach to improving the transparency of recommendation algorithms on social media that uses agent-based modelling. We motivate the need for sociotechnical transparency and demonstrate a novel application of agent-based modelling to this challenge. Our model will provide an insight into which curation signals the recommendation algorithm is prioritising at a high-level. Armed with this understanding, policymakers and the public can explore whether this aligns with what they think these algorithms are prioritising, whether this aligns with their values, and consider what they think it should prioritise.

### Challenges with transparency

There are a number of challenges facing approaches to transparency of recommendation algorithms on social media. We identify six key challenges, based on the survey presented in Sect. 2.2.1:

1. *Grounding*: Approaches, such as existing simulations, often have limited grounding in the system of interest due to limited or lack of access to data from the social media platform.
2. *Interactions*: Model-centric approaches are focused on the technical aspects of the algorithm and often do not account for interactions between the recommendation algorithm, the users, and environment.
3. *Variable Isolation*: On-platform studies have difficulty isolating variables in the real system due to its complexity. The whole system is heavily coupled therefore a change in the algorithm can result in unexpected changes elsewhere. This makes linking correlation and causation challenging.
4. *Scenario-Testing*: Approaches often can only assess the current state of the platform and “what is”. They do not enable scenario testing to predict the result of changes to the system. This is particularly useful for design changes or policy recommendations.
5. *Scale*: Approaches are often small scale, either by using a limited number of puppet accounts for on-platform experiments or a limited number of agents in simulated approaches.
6. *Platform-led*: Some approaches are platform-led, meaning they do not enable external oversight and do not necessarily align with what external stakeholders want to understand. External transparency approaches to transparency are critical.

Our proposed approach, using agent-based modelling, is designed to address these outlined challenges. Agent-based models can be grounded in the real system through data calibration, they can model complex systems with interactions, provide a controlled setting in which variables can be isolated and changed, they enable scenario-testing, they can be run with a large number of agents, and they do not rely on platform access [9, 10].

### Contributions

In this paper, we introduce the concept of sociotechnical transparency to highlight the importance of transparency that accounts for the interactions between the algorithm, users, and information. We will define and motivate the concept in Sect. 3. This paper demonstrates a novel application of agent-based models to improve the transparency of recommendation algorithms on social media platforms. Our model addresses the six key challenges presented by existing transparency approaches.

This research builds on work by Gausen et al. [9] that models the impact of different recommendation algorithm objectives on the spread of information and polarization on social media, using agent-based modelling. This paper extends the agent-based model to capture how the recommendation algorithm prioritises different curation signals. This moves away from counterfactual analysis to provide transparency on how the actual recommendation algorithm curates content. The contributions of this paper are:

1. To introduce the concept of sociotechnical transparency of recommendation algorithms on social media.
2. To present a novel approach to sociotechnical transparency using agent-based modelling. This approach provides transparency into how the recommendation algorithm prioritises different curation signals for a topic, at a high-level.
3. To develop an agent-based model of a social media platform with a novel implementation of a multi-objective recommendation algorithm that is calibrated and empirically validated with real data. Our model is based on the platform X, previously Twitter. This model achieves higher accuracy than previous models [9, 11] whilst encompassing higher complexity.

Our model is intended to address the following research questions:

1. Is the model able to capture the real system behaviour?
2. How does the recommendation algorithm differently weigh each curation signal across topics?
3. Are there consistencies in how the recommendation algorithm weights the curation signals across topics?

### Paper organisation

This paper is structured as follows: Sect. 2 provides context in terms of recommendation algorithms and regulation, and surveys existing approaches to transparency and agent-based models of social networks. Section 3 introduces the concept of sociotechnical transparency and outlines our proposed approach. Section 4 describes the design of the agent-based model and the recommendation algorithm implementation. Section 5 outlines the methodology, including the simulation pipeline, the evaluation metrics, the three datasets, and the experimental set-up. Section 6 presents the simulation results for each dataset. These are discussed in relation to the three research questions in Sect. 7. The limitations and implications of the approach are also explored. Finally, Sect. 8 concludes the paper and discusses directions for future work.

## Background

### Context

#### Recommendation algorithms

Social media platforms are made up a of number of different algorithms. These can be classified as *content processing* algorithms (such as language translation, annotation, etc) and *content proposal* algorithms (such as recommendation, search, etc) [12]. All these algorithms play an important role in the ecosystem of a social media platform. However, in this paper we focus on the content recommendation algorithms which curate and generate the newsfeed. This focus was chosen as they promote the greatest fraction of engagement [12] and it is the aspect platforms have the greatest control over. It important to note that even the “recommendation algorithm” itself can be made up of a number of different algorithms [13]. However, they are tightly coupled so we will treat them as a single entity, as in [12].

At a high-level, the recommendation algorithm is tasked with deciding what content to feed a specific user at a given point in time. It should rank this content based on the predicted likelihood that a given user will engage with it. This means that a core part of the algorithm is engagement. This is because user engagement promotes the macro-level objectives of the platforms such as revenue and user-base. The metrics to predict engagement will depend on the platform and content-type [12]. In our paper we do not focus on engagement metrics but instead on the curation signals that inform recommendation at a high-level.

#### Related regulation

There are a number of regulations emerging globally that will impact social media platforms. Current legislative proposals tend to focus on speech, such as the First Amendment, privacy, such as EU’s General Data Protection Regulation (GDPR) [14], and antitrust and competition, such as Clayton and Sherman Antitrust Acts in US [15]. Some scholars believe that privacy and antitrust regulation could be the most effective approach, as it bypasses imposing restrictions over content by instead offering users more agency over their data and choice of recommendation algorithm [16].

There is emerging regulation that will impact transparency of the recommendation algorithms on social media. We will provide a high-level overview. In the US, there are number of bills targeting algorithmic transparency [16]: S. 2024 Filter Bubble Transparency Act [17], H.R. 5596 Justice Against Malicious Algorithms Act [18], Algorithmic Accountability Act [19, 20], and Platform Accountability and Transparency Act [21]. These bills differ in the detail, but at a high-level they target algorithmic amplification, enable users to decide whether they are subjected to personalised curation, and require companies to assess the impacts of their systems. The UK Government has the Online Safety Bill [22]. This bill is not focused on individual posts but focused on forcing platforms to commit to their “promises”. Platforms will have to explain how they will deal with each type of harmful content in their terms of service. The UK have also published their whitepaper for future AI regulation [23], which cites social media algorithms are damaging to mental health [24].

The EU has the Digital Services Act (DSA) and Digital Markets Act (DMA) [25]. In terms of recommendation algorithms, a key part of these acts is to enable users to switch on personalised recommendation. The EU also has the Artificial Intelligence Act (AIA) which proposes risk-based regulation, where recommendation algorithms will categorised as “high risk" [26]. The EU Parliament reached a provisional agreement on the AIA at the end of 2023 [27], which included agreed upon obligations for “high risk" systems. This is seen as a significant step towards robust regulation of AI systems.

This overview of current and emerging legislation highlights public and governmental interest in social media platforms and their recommendation algorithms. The emerging regulation presents a positive step, however critics have highlighted that, with our current level of platform-led disclosures and our current external toolkit for transparency, much of the regulation will be very challenging to implement [28]. This motivates the need for novel tools for external transparency.

### Related work

#### Approaches to transparency

Currently, there are a number of approaches to transparency of recommendation algorithms on social media. There are both internal approaches, carried out by platforms themselves, and external approaches. This review will be based on the reviews by Bengani et al. [29] and Thorburn et al. [30].

In terms of internal, platform-led approaches, these range from documentation to high-level statistics to publishing privacy-protected datasets [29]. System-level documentation can include transparency reports, explanations of safety initiatives or high-level requests from government. Documentation, or explanations, can also be tailored to individual users, such as, transparent recommendation settings like Facebook’s “Why am I seeing this?” feature [31]. As both system-level and user-specific documentation is curated by the platform [32], this does not enable external oversight. Data approaches can involve API access to platform data or published curated datasets. With data transparency, there is often a trade-off between privacy and transparency. Finally, platforms can open-source code or publish details of the code in academic papers [33]. This model-centric transparency provides detail on how their recommendation algorithms work from an engineering perspective but these details are often different to what is important to policy-makers and the public.

There are a number of external approaches to transparency, which vary depending on the level of access to platforms and their data [30]. Recommendation algorithms are particularly hard to study in this context as classically transparency in the field of AI is focused on assessing training data for bias, representativeness and other metrics. However, this is aimed at classification tasks and recommendation algorithms do not have definable training data [34]. Without access to the platform or data, researchers are limited to simulations and off-platform studies. Simulation can lack grounding in the real system and off-platform studies tend to be small scale, such studies using puppet accounts [3]. The second group of approaches are for stakeholders with access to platform data, such as observational studies to identify correlations [35]. It is, however, challenging to link correlation and causation. Finally, external researchers with access to platforms can carry out on-platform experiments. Even in this case, it can be difficult to isolate variables to study, the whole system is heavily coupled and a change in the algorithm can result in unexpected changes elsewhere [30].

Overall, this review highlights that there are challenges with both internal and external approaches to transparency. In Table 1, we evaluate whether the reviewed approaches overcome six identified key challenges with transparency of recommendation algorithms on social media, initially outlined in Sect. 1.1: (C1) Grounding; (C2) Interactions; (C3) Variable Isolation; (C4) Scenario-Testing; (C5) Scale; (C6) Platform-Led. Internal approaches naturally suffer from being led by platforms and therefore will not necessarily align with what external stakeholders, such as researchers and policy makers, want to understand. External transparency initiatives are inherently challenging due to limited access to the system of interest [34]. Our proposal of using an agent-based model calibrated with real data could address each of these challenges. Additionally, regulation to put pressure on platforms to share externally prescribed sets of data with researchers will improve the state of research in this field.

**Table 1:** Evaluating whether current transparency approaches overcome the six challenges: (C1) Grounding; (C2) Interactions; (C3) Variable Isolation; (C4) Scenario-Testing; (C5) Scale; (C6) Platform-Led

	Approach	E.g.	C1	C2	C3	C4	C5	C6
Internal	Documentation	[36]	Y	Y	Y		Y	
	Datasets	[37]	Y	Y	Y		Y	
	Features	[31]	Y	Y	Y		Y	
	Open-source code	[33]	Y		Y		Y	
External	Off-platform studies	[3]		Y	Y			Y
	On-platform experiments	[38]	Y	Y		Y	Y	
	Observational studies	[35]	Y	Y			Y	Y
	Simulation without data	[39]		Y	Y	Y	Y	Y
	Proposed approach	–	Y	Y	Y	Y	Y	Y

#### Agent-based models

In this paper, we want to demonstrate that agent-based models could be a useful approach to provide transparency of the recommendation algorithms on social media. Agent-based modelling and simulation have been used in prior research to study social media networks. In order for them to be an effective approach to transparency they must overcome the six identified transparency challenges outlined in Sect. 2.2.1. The model will need to have the following properties (P1–P6) to overcome each of the challenges (C1–C6) of the same number: (P1) Be calibrated with real data; (P2) Model interactions between a recommendation algorithm, users, and information; (P3) Ability to change variables in the system; (P4) Enable scenario-testing; (P5) Be large scale (number of agents greater than 1,000); (P6) Enable external transparency of the real recommendation system behaviour. In Table 2, we review existing research that uses agent-based models to model social media networks, in relation to these six properties, to understand how well they address the transparency challenges and whether they could be used for this application.

**Table 2:** Review of existing agent-based models in relation to the six properties (P1–P6) required to overcome the transparency challenges (C1–C6)

Related work	P1	P2	P3	P4	P5	P6
Al Atiqi [40]		Y	Y	Y	Y	
Alassad et al. [41]	Y		Y	Y	Y	
Aridor et al. [42]		Y	Y	Y		
Chaney et al. [43]		Y	Y	Y		
Gausen et al. [11]	Y		Y	Y	Y	
Gausen et al. [9]	Y	Y	Y	Y		
Jiang et al. [44]		Y	Y	Y		
Kozitsin and Chkhartishvili [45]			Y	Y		
Murić et al. [10]	Y		Y	Y	Y	
Onuchowska and Berndt [46]			Y	Y		
Fränken and Pilditch [39]			Y	Y		
Proposed model	Y	Y	Y	Y	Y	Y

DARPA’s Computational Simulation of Online Social Behavior (SocialSim) is a significant research effort in this space in recent years. This aimed to develop novel computational simulations of online behaviour, specifically focused on information propagation on three platforms: Twitter, Reddit and Github [10, 47]. Muric et al. [10] present agent-based models where the agents’ decision workflow use machine learning. The focus of this research project was on information propagation on platforms, not on recommendation algorithms. However, this project highlights the importance of using real data when simulating behaviour.

A number of papers use agent-based models to model the spread of misinformation [11, 46] or malicious information [41]. Other papers use agent-based models to model the formation of echo chambers [39, 40, 45]. There are examples of agent-based models that model the recommendation algorithms on social media. Some papers study the emergence of filter bubbles from recommendation algorithms homogenising the content users are exposed to [42, 43]. Jiang et al. [44] model both recommendation algorithm behaviour and user dynamics, to separate the effects of filter bubbles and echo chambers. Gausen et al. [9] use agent-based modelling to understand how varying the objective of the recommendation algorithm impacts the propagation of information and echo chamber formation online. This research is counterfactual, for transparency we need to understand how the actual system is working. Based on this review, our proposed model will be novel in having all six properties required to address the key transparency challenges, presented in Sects. 1.1 and 2.2.1.

## Introducing sociotechnical transparency

### Why is sociotechnical transparency important?

In this section we will first motivate the need for improved transparency of recommendation algorithms on social media, then present why considering the sociotechnical nature of these systems is critical for meaningful transparency for the public and policy-makers.

Social media platforms have changed who has the power to create content, how content is created and how it propagates [1]. There have been many criticisms of the impact of social media platforms and their recommendation algorithms, from rising hate speech to political disinformation campaigns. Faced with rapid advancements in generative AI capabilities, many are concerned that these risks will be amplified further [48]. In a pivotal case in 2022, the death of a teenage girl Molly Russel was attributed to social media algorithms in a coroners report. This significant delegation of responsibility to the platforms highlighted the impact of these algorithms. Some call for the return to a non-algorithmic, reverse chronological newsfeed. However this will result in a random selection based on time [1]. Others call for improved transparency in the sector [7, 49–51]. These researchers and advocates want to understand: what is the algorithm doing now and what alternatives could there be? Their call for transparency will be bolstered by new regulation, including UK Online Safety Bill, EU AI Act, EU Digital Services Act and the Algorithmic Accountability Act.

Transparency is the first step towards understanding the societal implications of social media, enforcing regulation and performing external audits [7]. This transparency should have a significant focus on the recommendation algorithms. The algorithms are an aspect of the social networks that platforms have control over; they cannot be responsible for individual pieces of content with the scale of what is on social media but they should be responsible for what their algorithms promote and amplify [49, 50]. Interestingly, many social media platforms discuss their algorithms openly in academic papers [36, 52, 53] and some have even published parts of their codebase [33]. This model-centric transparency is useful from an engineering perspective and for determining technical functionality [54]. However, these disclosures are not sufficient for the public and policy-makers to understand the implications of these algorithms on society [55]. This has resulted in the “current unsatisfactory and somewhat paradoxical state of algorithmic transparency" [12].

In this paper, we present the concept of socio-technical transparency, which can be defined as transparency approaches that account for both the technical system, and how it interacts with users and the environment in which it is deployed. Algorithms are “technical constructs that are simultaneously deeply social and cultural" [56]. If transparency is bounded to just consider the recommendation system in isolation, this abstracts away the social context in which the system is deployed within and entangled with [57]. It is the interaction between both the social and technical components that dictates risk from a system [58]. We propose that this type of transparency will provide more meaningful understanding for policy-makers and the public.

### Using agent-based modelling for sociotechnical transparency

In this paper, we propose an approach to sociotechnical transparency of recommendation algorithms on social media. Our survey, highlighted the importance of building tools for external transparency [34] and the challenges with current approaches. We propose a novel approach that uses agent-based modelling calibrated with real data that addresses these challenges.

Prior research has shown agent-based models can capture complex sociotechnical phenomenon and provide insight into the impact of the recommendation algorithm objectives [9]. They sit between theoretical and empirical approaches [59], enabling the study of recommendation algorithms without the ethical concerns that exist with empirical longitudinal studies. Agent-based models calibrated with real data overcome the six key challenges (C1 - C6), identified in Sect. 2.2.1: they can be grounded in the real system through data calibration (C1), have the ability to capture the “emergent effects of human-algorithm interactions” [12] and “underlying feedback loops” [12] (C2), they provide a very controlled setting so variables can be isolated and changed (C3), they enable “what-if” analysis (C4), they enable experimentation with a large number of agents (C5), and they do not rely on direct access to the platform (C6). However, the application of agent-based models to this problem is novel and, unlike existing research, our proposed model will address all six transparency challenges. This comparison with existing agent-based models can be found in Table 2 in Sect. 2.2.2.

Our approach aims to provide one type of sociotechnical transparency: an insight into which curation signals the recommendation algorithm is prioritising at a high-level. This is not the same as the engagement metrics discussed in Sect. 2.1.1. This model accounts for the interactions between the recommendation algorithm, the users, and the network. This type of transparency can provide insight for regulation, such as the Online Safety Bill [22], which are designed not to penalise based on individual pieces of content but to ascertain whether the platforms are doing what they claim to be doing. We can begin to explore whether the priorities of their recommendation algorithm aligns with what platforms say they are doing and whether it aligns with what the public want.

### Considerations

Our approach hopes to offer meaningful transparency about recommendation algorithms on social media using agent-based modelling, that accounts for user and network interactions. It is important to highlight some considerations with this approach.

Firstly, the overview in Sect. 2.2.1 outlined the challenges faced by transparency approaches in this space. Our approach addresses these challenges however it still faces the same barriers that the outlined external approaches encounter in terms of limited platform and data access. For example, data on what appears on an individual user’s newsfeed is not public therefore we must simulate this based on retweet data that we can collect.

Secondly, agent-based modelling and simulation have limitations. These types of approaches can be criticised for lacking grounding in the real system and having simplified models of behaviour that incorporate many assumptions. As a wider field, agent-based models and simulation would benefit from more standardisation and common conceptualisations [59]. It is beyond the scope of this paper to tackle these issues but we do ensure our model is grounded in real data, has a significant number of agents, and is validated using a set of evaluation metrics [10].

Thirdly, for this approach the agent-based model itself must be based on a specific social media platform. This means that the agent-based model will not be generalizable across platforms unless they have the same information sharing mechanisms and follower/followee structure. However, the high-level approach could be generalized across platforms, including the calibration method and the weight optimisation process, provided that an agent-based model of that platform has been developed and there is available data on information propagation.

Despite these considerations, our approach still represents an advancement in terms of sociotechnical transparency using agent-based modelling. Whilst external transparency of recommendation algorithms on social media is challenging, it is important to develop new tools to improve policy-makers’ and the public’s understanding of these algorithms behaviour. This research presents a first step towards a novel approach, which means that it is not mature enough to be used for auditing or regulation. However, we hope this will be an important proof of concept that will motivate further research into developing a more formalised tools.

## Proposed model

The aim of this research is to improve transparency of how the recommendation algorithm prioritises different curation signals. This approach uses an agent-based model of X, previously known as Twitter, and is calibrated with real data. This section describes the proposed agent-based model and the recommendation algorithm implementation.

Our model is novel in its ability to provide transparency of the curation signals prioritised by a recommendation algorithm on social media, based on the survey in Sect. 2.2.2. This paper extends the model presented by Gausen et al. [9]. The novelty of our research is the recommendation algorithm design and the simulation pipeline enable our model to capture the actual behaviour of the recommendation algorithm being studied. Whilst the previous work [9] could only run counterfactual experiments where the recommendation algorithm behaviour is user specified.

This is enabled by two significant developments. Firstly, the recommendation algorithm design is extended. In the previous work [9], the recommendation algorithm could be either chronological, belief-based, popularity-based, or random. In this research, our model curates the newsfeed based all four signals, where their influence is controlled by a set of weightings. More information can be found in Sect. 4.2. Secondly, the previous research [9] required the user to specify the behaviour of the recommendation algorithm as one of either chronological, belief-based, popularity-based, or random. This research develops an optimisation algorithm that finds the weights of each curation signal that best reflects the behaviour in the real data. More information can be found in Sect. 5.1. This optimisation occurs across multiple tweets, whilst previous work only analysed the propagation of a single tweet [9].

### Agent-based model design

This paper introduces a novel agent-based model based on the social network X, where the agents represent individual users on social media. This model extends the work by Gausen et al [9]. The connections between agents symbolize follower/ followee relationships, and agents can access information posted as tweets shared by their network connections. The inspiration behind this model stems from epidemiology modeling, drawing parallels between the dissemination of information on a social network and the spread of a disease in a population [60, 61].

The model focuses on the two primary mechanisms for information propagation on X, which are the retweet functionality and the recommendation algorithm [10]. The retweet functionality allows users to re-share tweets they see on their newsfeed resulting in that tweet propagating through the network. The retweet propagation is recorded in API data, which enables model calibration and validation. The content visible to users on their newsfeeds is controlled by a recommendation algorithm model, discussed more in Sect. 4.2.

**Fig. 1:**
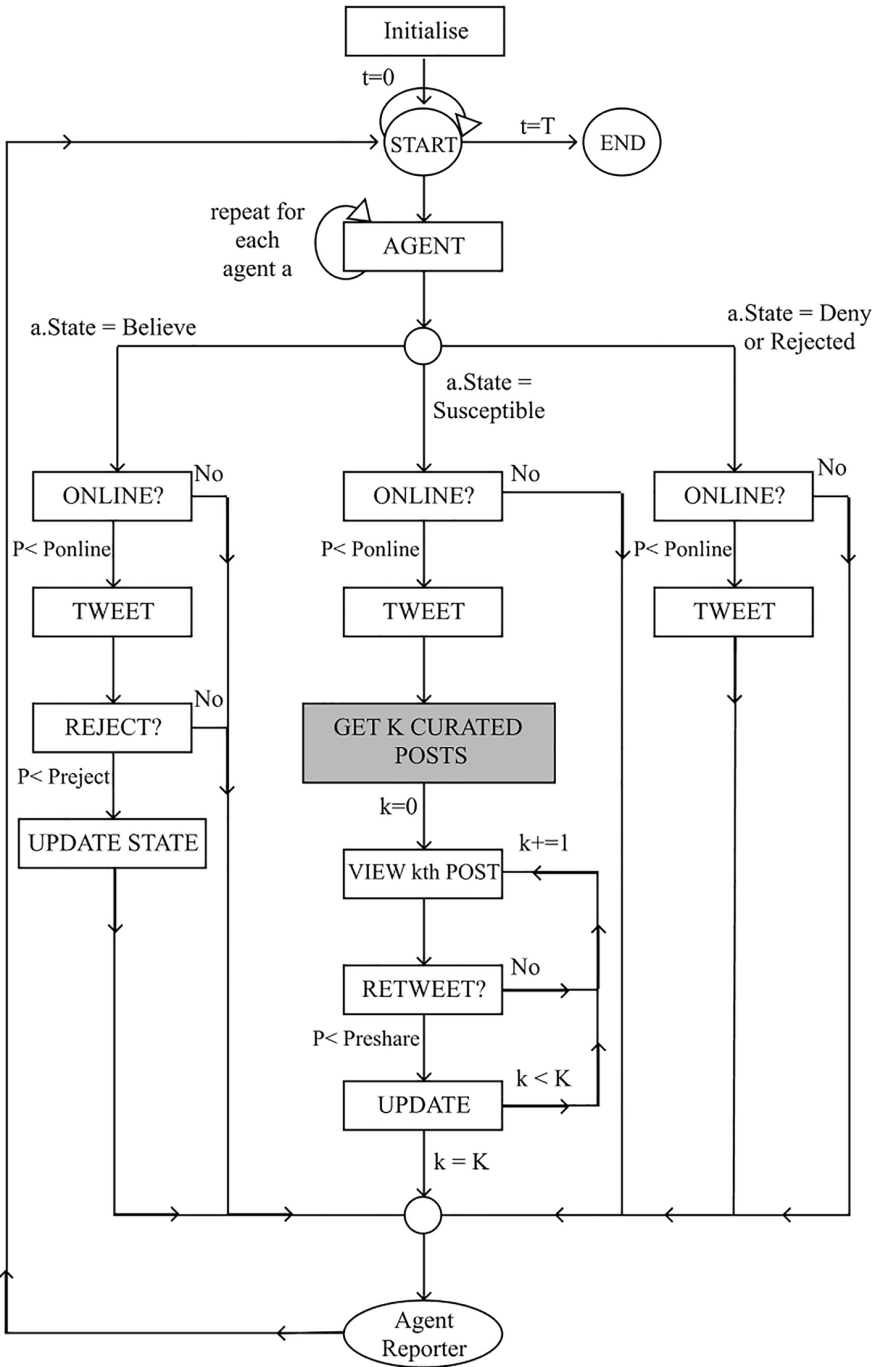
Diagram describing proposed model logic

Figure 1 provides an overview of how the model has been programmed. The information propagation is captured by the tweet and retweet functions whilst the logic of the recommendation algorithm is represented by the “get curated posts” box. Each run of the agent-based simulation is focused on tracking the propagation of a single tweet for *T* timesteps. Therefore the agents’ state correspond to whether they believe (i.e. retweet/tweet the story), are susceptible (i.e. not yet rejected/retweet the story) or reject and deny (i.e. have rejected the story). The model behaviour is controlled by three probability distributions: (1) $P_{\text {reshare}}$: the probability that an agent retweets the story. (2) $P_{\text {reject}}$: the probability an agent rejects the story and will not retweet it in the future. (3) $P_{\text {online}}$: the probability that an agent is online in a given timestep. The first two probabilities are calibrated with data collected on the retweet propagation of a story, see Sect. 5.3, and the mean of the probability that an agent is online was set as the mean value used in [9].

In each timestep, agents sample probabilities to determine whether they are online and, if so, whether they will tweet. If an agent is online and views their newsfeed, they will read the *K* top curated posts from their neighborhood based on the curation signals. For each post they view, they will sample a probability distribution to determine whether they retweet it. If an agent tweets, it will be based on their current beliefs, and if they retweet a post it will be incorporated into their beliefs and could impact their state. This logic is applied only to agents who are susceptible to the story. The agent reporter records the agent states and retweets at each timestep.

In our model, one of the curation signals for the recommendation algorithm is belief-based. To model beliefs, our model is populated with Bayesian agents. Bayesian agents are agents with Bayesian belief updating, which is when the beliefs of the agents update “perfectly” when faced with new information. This means that agents integrate new information, from posts that they view, to their existing beliefs without cognitive biases [62]. This type of belief updating is modelled by the following equations. The probability of an agent’s beliefs given new evidence is [9, 63]:

1 $$\begin{aligned} P(H|E) = P(H) * P(E|H)/P(E) \end{aligned}$$

where *P*(*H*) is the agent’s original belief and *P*(*E*) is the probability of the evidence regardless of the agent’s own beliefs, which is calculated as:

2 $$\begin{aligned} P(E) = \frac{1}{\sigma _{T}*\sqrt{2 \pi }} * e^{\frac{(X-\mu _T)^2}{2 \sigma _{T}^2}} \end{aligned}$$

where $\mu _T$ is the mean of the true distribution, $\sigma _{T}$ is the standard deviation, and *X* is the new evidence observed by the agent. In our model, the evidence *X* is the belief of a neighbouring agent whose post the agents views, where $0 \leqslant X \leqslant 1$. Finally, the probability of that evidence accounting for the agents’ own beliefs is calculated by:

3 $$\begin{aligned} P(E|H) = \frac{1}{\sigma _{A}*\sqrt{2 \pi }} * e^{\frac{(X-\mu _A)^2}{2 \sigma _{A}^2}} \end{aligned}$$

where $\mu _A$ is the mean of the agents own belief distribution, and $\sigma _{A}$ is the standard deviation. It should be noted that it is very common for Gaussian distributions to be used for Bayesian belief updating [62]. This is because they provide favourable mathematical properties and the central limit theorem, which is a statistical premise that as the size of a sample gets sufficiently large, the distribution of a variable from that sample will approximate a normal distribution, regardless of the original distribution [64].

### Recommendation algorithm implementation

It is critical to highlight that this research is not trying to directly mimic the recommendation algorithm that curates newsfeeds on social media, but instead provide insight into which high-level curation signals it prioritises, using simulation and real data.

A recommendation system provides an underlying score to estimate how likely a user will engage with a given post at a given time. Given the set of scores for potential posts, the recommendation algorithm ranks them and the user views the top *K* posts, where *K* is sampled from a distribution. The value $K_{mean}$ comes from empirical user behaviour data [65] and the standard deviation of the distribution is set to $K_{mean}/2$. In this proposal, the score accounts for four curation signals: chronological, belief, popularity, and random [9, 43]. The recommendation algorithm curates the tweet based on weightings of each curation signal. The weightings of each signal, i.e. how significantly the algorithm prioritises that signal, is found from the weight optimisation part of the pipeline that tries to approximate what is happening in the real data.

For the curation signals, the following logic is used to find the ranking for each signal. For chronological ranking, the posts are ranked by the most recently posted. For a belief-based ranking, the posts are ranked by the beliefs of the users that are most aligned to the agent. For popularity-based ranking, the posts are ranked in order based on their popularity. Finally, random ranking is just a random shuffle of the posts. These individual curation signals feed into the curated newsfeed using the weightings of each signal $\left[ w_0, w_1, w_2, w_3\right]$. Once ranked, the top *K* posts are surfaced to the agent. This logic is outlined in Algorithm 1.

**Algorithm 1:**
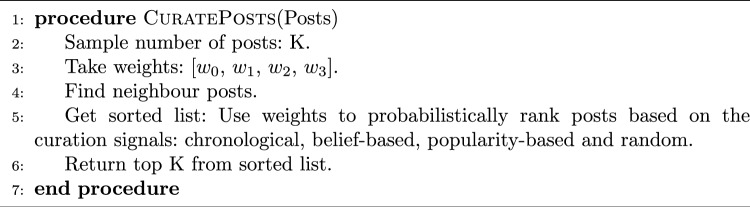
Recommendation Algorithm

## Methodology

This section describes the methodology used for this research, including the simulation pipeline, evaluation metrics, data, and the experimental set up for the results presented in Sect. 6.

### Simulation pipeline

**Fig. 2:**
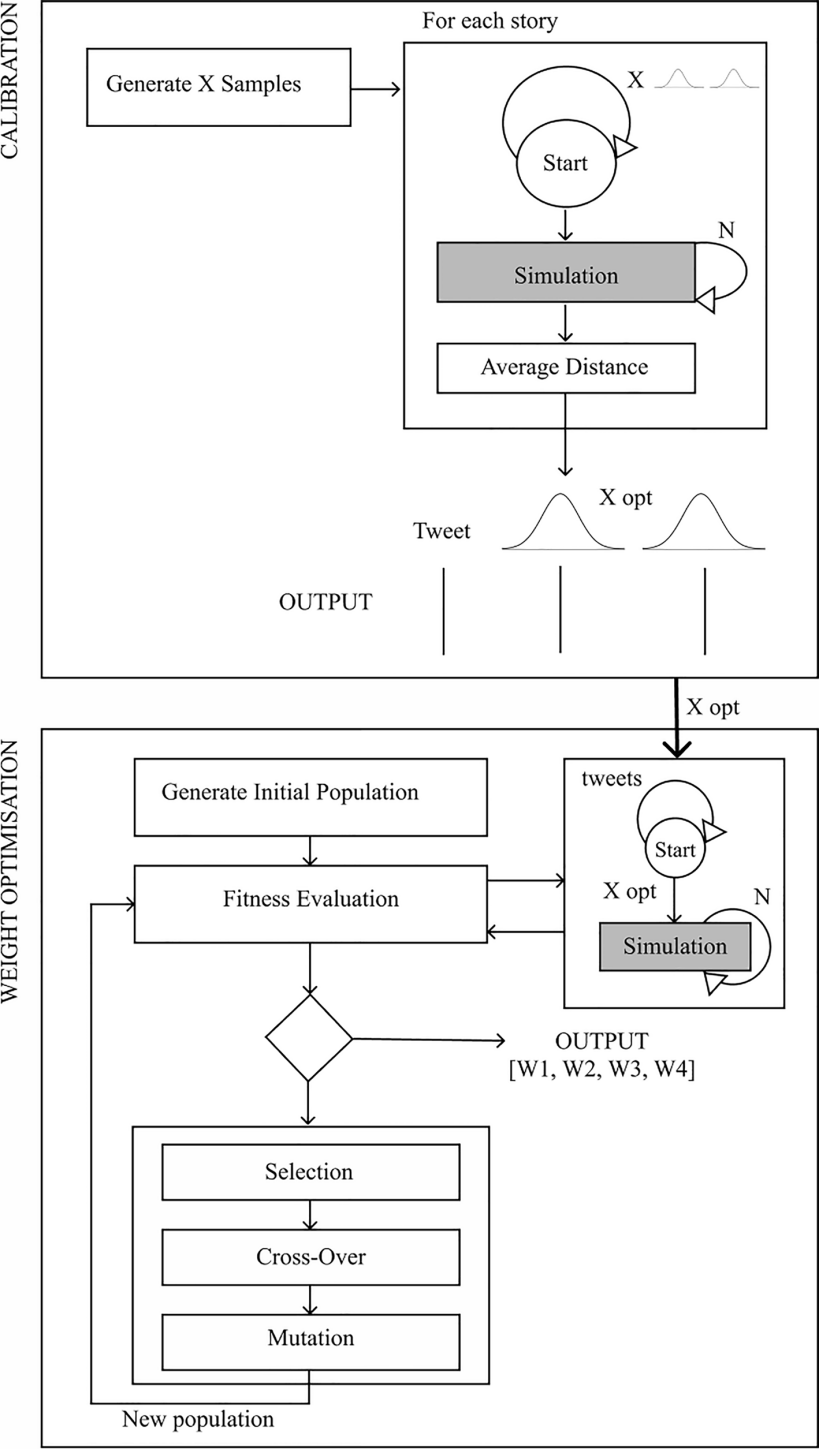
Diagram outlining the simulation pipeline

The simulation pipeline is the process that takes a dataset of real tweets as input and outputs an estimate of the weightings of the four different curation signals for recommendation. The simulation parameters used for the experiments are presented in Sect. 5.4. Figure 2 provides an overview of the pipeline. The first stage is calibration, in which the probabilities that govern the agent-based model are calibrated to the real tweets in the dataset. The calibration process calculates the probability distributions that govern the retweet behaviour for each tweet in the dataset. This then acts as the input to the weight optimisation stage. A genetic algorithm (GA) is used to estimate the high-level weightings of each curation signal in the recommendation system based on the datasets, see Algorithm 11 for more details. GAs are an evolutionary algorithm that can be used for optimization tasks. This was chosen for its ability to reach good solutions with limited runs of the simulation [66]. Here the fitness function is trying to minimise the distance between the real and simulated data in each timestep across all the tweets within the dataset for candidate weightings. For each set of candidate weightings, the simulation is run *N* times for all tweets in the dataset to calculate the fitness function for that candidate. The optimum candidate is the set of weights that control the behaviour of the recommendation algorithm so that the simulated data best matches the real behaviour, across all tweets in the dataset. For the experiments, we used a value of *N = 5*, for justification see Appendix A.1. The output of this stage is an optimal set of four weights, which corresponds to how the recommendation algorithm weighs each curation signal, based on a dataset.

**Algorithm 2:**
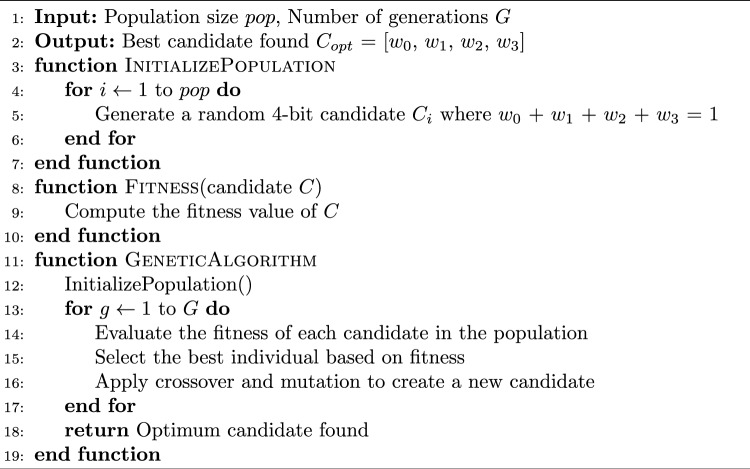
Genetic Algorithm

### Evaluation metrics

It is critical to evaluate that the simulation is able to capture the real system behaviour. In order to evaluate this we carry out three evaluations [10]. Firstly, we evaluate that there is correlation in the speed of retweets between the simulated output and real data. This is calculated as the root-mean-square-error $RMSE_{v}$ in retweets in a given timestep between the real data ($y_i$) and simulated data (${\hat{y}}_i$). It should be noted that the number of retweets ($y_i$, ${\hat{y}}_i$) are not absolute values. They are proportions, calculated as the ratio between the number of retweets (per a timestep) and the population size in the simulated or real data. This process is required to scale the simulation to the real data, as the real system will have much greater numbers of users and connections. The real population proxy calculation was taken from [9] as the ratio between the simulated population and average node degree multiplied by the average number of followers in the dataset. The equation for $RMSE_{v}$ is:

4 $$\begin{aligned} RMSE_{v} = \sqrt{\frac{1}{\tau } \sum _{i=1}^{\tau } (y_i - {\hat{y}}_i)^2} \end{aligned}$$

where $\tau$ is the total number of data points, $y_i$ represents the real data at point $i$, ${\hat{y}}_i$ represents the simulated (predicted) data at point $i$, and the summation $\sum _{i=1}^{\tau }$ is taken over all data points. Then we evaluate that there is correlation in the total number of retweets between the simulated output and real data. This is calculated as the *NRMSE* as in Equation 6, where the $RMSE_{n}$ is the difference between the total number of tweets in each story between the real and simulated data:

5 $$\begin{aligned} RMSE_{n} = \sqrt{\frac{1}{\tau } \sum _{i=1}^{\tau } (r_i - {\hat{r}}_i)^2} \end{aligned}$$

where $\tau$ is the total number of tweets in the dataset, $r_i$ represents the real number of retweets for tweet $i$, ${\hat{r}}_i$ represents the simulated (predicted) number of retweets for tweet $i$, and the summation $\sum _{i=1}^{N}$ is taken over all tweets in the dataset. Both the metrics are normalised (NRMSE):

6 $$\begin{aligned} NRMSE = \frac{RMSE}{\Phi _{max}-\Phi _{min}} \end{aligned}$$

where $\Phi _{max}$ and $\Phi _{min}$ are the maximum and minimum of the proportion of tweets across all time steps in a simulation. Finally, we measure the similarity in the distribution of data between the simulated and real. This was calculated using the Jensen-Shannon (J-S) divergence. The J-S divergence is calculated as:

7 $$\begin{aligned} JSD(P \parallel Q) = \frac{1}{2} \left( D_{KL}(P \parallel M) + D_{KL}(Q \parallel M) \right) \end{aligned}$$

where $JSD(P \parallel Q)$ is the Jensen-Shannon Divergence between the probability distributions $P$ and $Q$, $D_{KL}(P \parallel M)$ is the Kullback–Leibler Divergence between $P$ and the midpoint distribution $M$, $D_{KL}(Q \parallel M)$ is the Kullback–Leibler Divergence between $Q$ and the midpoint distribution $M$, and $M$ is the midpoint distribution. This was calculated for each tweet in a dataset then averaged for that dataset.

### Data

The simulation pipeline was run for three datasets. The data collection used the Twitter Academic API. It should be noted that the Academic API access has been removed since the collection date. However, there are new data sharing initiatives that could be leveraged, such as X API access under Article 40 of the Digital Services Act [67]. With API access, the collection method should be the same as it was for the collection of this data.

We present results for three different datasets: “Turkey Earthquake”, “Brits 2023” and “Balloon Incident”. Each dataset represents a set of tweets collected on given topic of interest at the time of collection, February 2023. The topics were chosen based on the trending topics function. Once a topic was chosen, tweets containing related keywords and hashtags were collected with their corresponding retweet history. The properties of each dataset can be found in Table 3.

**Table 3:** Dataset properties

Parameters	Dataset		
	Turkey Earthquake	Brits 2023	Balloon Incident
Number of tweets	50	50	50
Number of retweets	6780	838	924
Average duration (h)	18.94	15.76	17.48
Dataset tweet start date	06/02/2023 01:19	02/02/2023 12:02	05/02/2023 05:52
Dataset tweet end date	06/02/2023 06:17	11/02/2023 17:04	10/02/2023 21:08
Dataset retweet start date	06/02/2023 01:23	02/02/2023 16:56	05/02/2023 05:56
Dataset retweet end date	11/02/2023 21:50	12/02/2023 09:07	14/02/2023 14:07
Collection keywords	Turkey + earthquake	Brits + 2023	US + balloons
Collection hashtags	Turkey + earthquake	Brits2023, Brits	BalloonShotDown
	TurkeyEarthquake	Brits + 2023	US + balloons

The datasets themselves were collected with a custom python script, using the Tweepy library. This handles authorization of the accesses token and calling the API. Once the API was verified, tweets were collected based on a topical set of hashtags and keywords over a date period. The hashtags, keywords, and date periods for each dataset are specified in Table 3. A short collection period was chosen because it is more useful when simulating temporal activity on social media, due to algorithmic variation and variations in the user population [47]. The original tweet information was collected for the tweet (tweet_id, created_at, no_retweets) and the user who posted the tweet (user_id, verified, followers_count). Then this was repeated for the retweets associated with that tweet.

Additionally, we use empirical user behaviour data to initialise certain model parameters. The average number of posts viewed by a user when online was estimated as 40 based on [65] and the retweet activity increase for verified users compared to non-verified users was calculated using the FakeNewsNet Dataset [9, 68].

### Experimental set-up

For the experimental set-up, each dataset is passed through the full simulation pipeline. This means that for each dataset, the probabilities that control the spread of each tweet is calibrated with the real data, the weights of curation signals are calculated across all tweets in the dataset then the simulated output is evaluated against the real data based on a set of metrics. The simulation parameters used for these experiments are presented in Table 4.

**Table 4:** Simulation parameter values

Simulation parameter	Value
Number of agents	1500
Node degree	250
Number of simulations (*N*)	5
GA population size	50
GA number of generations	10
Individual runs	50

The choice of the genetic algorithm population size, number of generations, and the total number of simulations are determined as the number required for convergence, see Appendix A.1. The number of agents and node degree are very impactful on simulation run time so it is not possible to have them set as realistic values. This is a common challenge in agent-based modelling. For our research, we set the number of agents and node degree to be sufficiently high to capture the values of the proportional number of retweets per a timestep $y_i$. For example, a population of 100 agents would only enable a granularity of $1\%$ and therefore wouldn’t sufficiently capture the behaviour seen in the dataset.

## Results

This section presents the results of running the simulation pipeline for the three datasets, outlined in Sect. 5.3. Table 5 presents the evaluation metrics for each set of results. These describe how closely the simulated output captures the behaviour in the real world data. The precise evaluation metrics used are described in detail in Sect. 5.2.

**Fig. 3:**
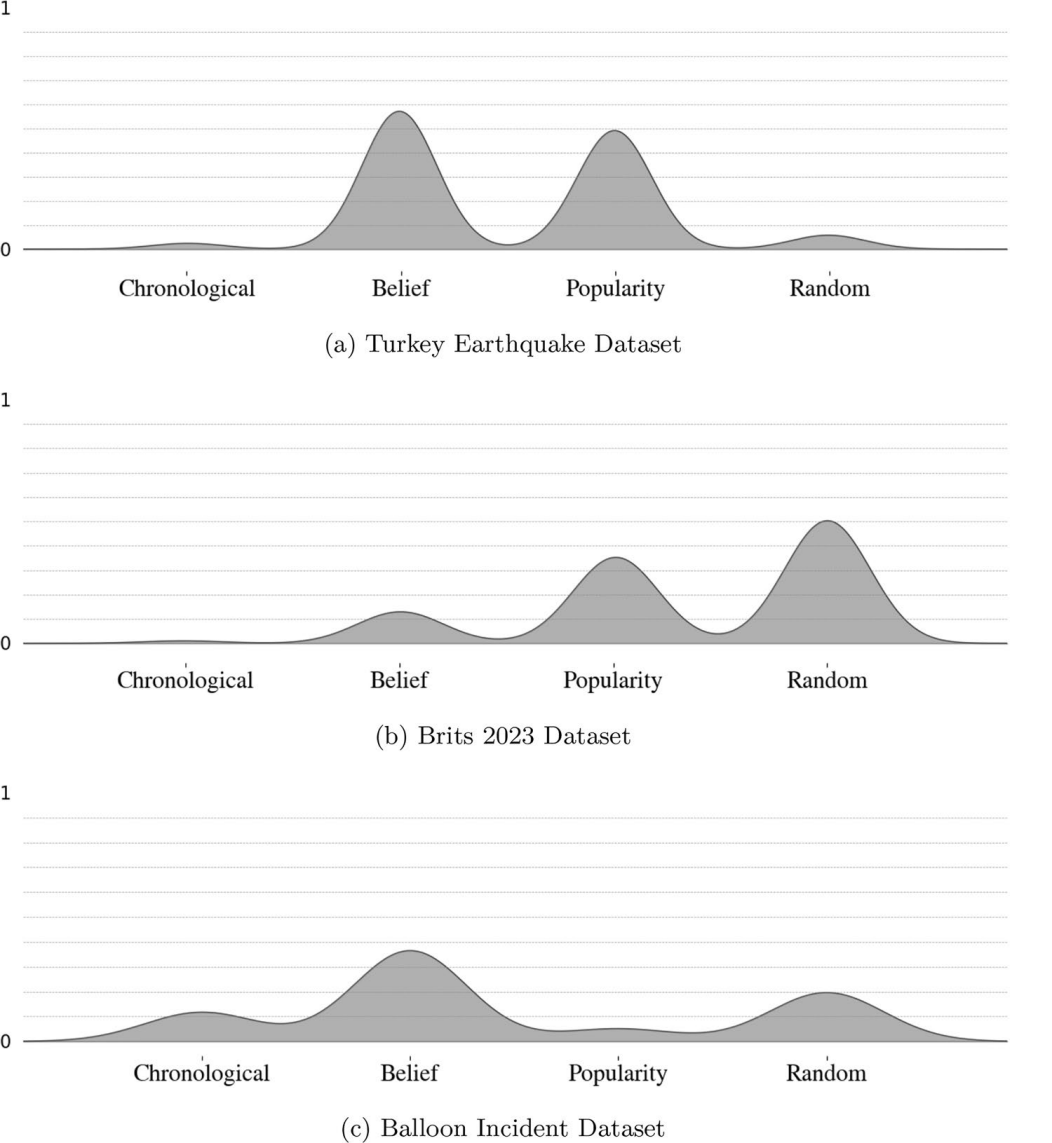
Results from the simulation pipeline: weights on curation signals for each dataset

Figure 3 presents the output of the simulation pipeline for all three datasets. The peaks of each curve represent the relative weightings of each of the curation signals: chronological, belief, popularity and random. The weighting values sit between 0 and 1. Figure 3a presents the final output of the simulation using the “Turkey Earthquake” dataset. Figure 3b shows the final output of the simulation using the “Brits 2023” dataset. Figure 3c shows the final output of the simulation for the “Balloon Incident” dataset.

**Table 5:** Evaluation metrics for each run

Evaluation metrics	Datasets		
	Turkey Earthquake	Brits 2023	Balloon Incident
Mean JS divergence	0.110	0.080	0.098
Standard deviation JS divergence	0.022	0.0185	0.020
Correlation of speed of retweets (RMSE)	0.00167	0.00175	0.00067
Correlation of speed of retweets (NRMSE)	0.0538	0.0854	0.0509
Correlation of total retweets (RMSE)	0	4.66E−15	1.11E−15
Correlation of total retweets (NRMSE)	0	2.28E-11	8.45E−12

## Discussion

We will discuss the results presented in Sect. 6 in relation to three key research questions. We will then explore how these results capture sociotechnical transparency and how they could create future analysis opportunities to help policy makers and the public make informed decisions about recommendation algorithms on social media. Finally, we will discuss current limitations.

### Research questions

#### Is the model able to capture the real system behaviour?

The first question aims to understand how well the model replicates the real system behaviour. This is the crucial step in validating our implementation and the proceeding analysis. In order to answer this question, we used a set of evaluation metrics. These metrics are described in Sect. 5.2 and presented in Table 5.

Firstly, we evaluate the correlation in the speed and volume of retweet propagation between the real data and the simulated output, as an average across all tweets. The value of the NRMSE across each dataset is in the range of 0.0509 to 0.0854. Our model outperforms previous models in terms of accuracy. The model results presented in Gausen et al. [9] had an NRMSE value of 0.25. Additionally, our model complexity is higher as it must capture behaviour across 50 tweet propagations simultaneously, whilst the previous paper was modelling a single tweet propagation [9].

Secondly, we evaluate the correlation between the total number of retweets for a given tweets between the real data and the simulated output, as an average across all tweets in a dataset. As can be seen in Table 5, this value is very well captured by the model. Finally, we evaluate how well the model captures the distribution of data in each dataset. We have calculated the Jensen-Shannon divergence for each dataset and the values are close to the threshold of *0.1*. This indicates that the model does capture the distribution of data [10].

The results of these three evaluation metrics indicate that our model is able to capture the behaviour observed in the real datasets, in terms of number of retweets, speed of retweets, and distribution of data.

#### How does the recommendation algorithm differently weigh each curation signal across topics?

The second question this research poses is how does the recommendation algorithm weigh the importance of each curation signal across topics.

We present the results for three different topics in Fig. 3. Figure 3a shows that for the topic of “Turkey Earthquake” the recommendation of content was primarily focused on the popularity of the content and belief-based signals. The weightings of these two signals are almost equal at 42.6% and 49.8% respectively. Whilst Fig. 3b shows that for the topic of “Brits 2023” the recommendation of content was split between three primary signals. The model output indicated the around 50% of the weighting was on random signals, around 35% was on popularity of content, and just over 10% was on belief. Finally, for the topic “Balloon Incident”, the recommendation of this content is primarily driven by shared belief with 50% of the weighting.

The figures clearly show that the recommendation algorithm weighs the curation signals differently for different topics. This difference could be due to the type of topic in the data. For example, the “Turkey Earthquake” and the “Balloons Incident” are both news stories whilst the Brits is popular culture event. It could be intuitive that news topics would be more belief-based than those related to popular culture.

#### Are there consistencies in how the recommendation algorithm weights the curation signals across topics?

The final research question we discuss is whether there are consistencies across topics in how the recommendation algorithm prioritises the curation signals. Across all three topics the recommendation algorithm has a very low weighting on chronological signals. This indicates that in a short time period, how recently a piece of content was posted, will not play a significant role in how high the algorithm will rank it within a user’s newsfeed.

### Limitations

The proposed approach has a number of considerations, discussed in Sect. 3.3. Here we will discuss the limitations of our implementation. Firstly, our model is based on one social media platform and it considers the recommendation algorithm system singularly [12]. Additionally, we are limited in the number of topics we analyse. Access to academic API for X has stopped. However, there are new data sharing initiatives that could be leveraged, such as X API access under Article 40 of the Digital Services Act [67]. In this research only four curation signals were implemented. This decision was based on existing literature [9] but in the future different signals could enrich the research. Finally, there are limitations in the size of our simulation. Due to the scale of social media platforms it is very difficult to model realistic populations. However, a population size of 1,500 agents is still higher than most reviewed literature.

Despite these limitations, our model was able to sufficiently answer the three research questions, discussed in Sect. 7.1. For question 1, the results of the evaluation metrics indicate that our model is able to capture the real system behaviours that the study is focussed on: number of retweets per a time step, total number of retweets, and the distribution of data. For question 2, our model was able to show that the recommendation algorithm differently weighs the curation signals across different topics. For question 3, the model showed that the algorithms consistently has a low weighting on chronological signals across topics. However, our collection period is short and we may see the chronological signals becoming more significant over a longer time period. This could be an interesting direction for future research.

### Wider analysis opportunities

In this section we have discussed the results in relation to three defined research questions. However, it is important to explain how these insights provide sociotechnical transparency, and how policymakers and the public could leverage them. Firstly, the results provide new insight into the curation signals that the recommendation algorithm uses to surface content to users for specific topics. This provides different information to model-centric transparency approaches, focusing less on how technically the algorithm curates content for users but instead on how it interacts with users and information on the platform. Secondly, these insights could be leveraged by policymakers to understand what signals the algorithm uses to recommend different types of information. This analysis could be applied to different harm types instead of topics to inform regulation, such as the UK’s Online Safety Bill. Alternatively, one could record additional metrics, such as, the prevalence of the information type appears on users’ simulated newsfeeds. This could help us understand if this aligns with what platforms say they are doing and whether this aligns with what the public would think was appropriate. Furthermore, this type of modelling could be used as a sandbox to test alternative recommendation algorithm designs and evaluate their impact on the spread of different types of content through the network. Interacting with policy makers could shape new research questions to explore with the agent-based model. These models are easily adaptable and offer a controlled setting for experimentation, making them well suited to support policy formulation and evaluation [69].

## Conclusion

Recommendation algorithms on social media are hugely impactful, they shape the flow of information and human connection at an unprecedented scale. Despite heightening criticism regarding their social impact, these algorithms remain largely opaque and transparency is challenging. In this paper, we introduce the concept of sociotechnical transparency. This is defined as approaches that account for how the algorithm interacts with users, information, and the environment in which it is deployed. Moving away from model-centric approaches should provide more meaningful transparency for policy-makers and the public.

In this paper we propose a novel approach to improving the transparency of how the recommendation algorithm prioritises different curation signals. This approach uses agent-based modelling to model the social network, X, and is curated with real data collected from the platform. The evaluation of the model validated that the agent-based model could capture the dynamics in the real data. The results show that the algorithm prioritises curation signals differently for different topics. This insight into how the algorithm curates for different topics will help inform discussions around whether this aligns with what platforms say and with what the public want, feeding into regulation of the sector. Since recommendation algorithms play such a central role in society, we propose that agent-based models should be included in a toolkit of external approaches to transparency of recommendation algorithms. These models can address many of the existing challenges and enhance our understanding.

The proposed approach has limitations, discussed in Sect. 7.2, which point to a number of avenues for future work. Firstly, this paper focuses on one social media platform and a dataset of three topics. Future work could expand this scope to look at more topics and model different platforms. This will require the ability to model the information propagation mechanism on the platform and access to data. Secondly, the population size used is not at the scale of a real social media platform. More work into acceleration could enable the analysis to be run for larger populations of agents. Thirdly, this paper presents a novel approach that is still in the proof-of-concept stage. In order for it to be used as a tool for sociotechnical transparency, further work should be carried out in formal verification of the results and in expanding the scope. Finally, this proposed approach was developed in response to the current state of algorithmic transparency. New regulation will hopefully lead to improved access to platform data and the algorithms, unlocking new approaches to sociotechnical transparency.

## Figures and Tables

**Fig. 4:**
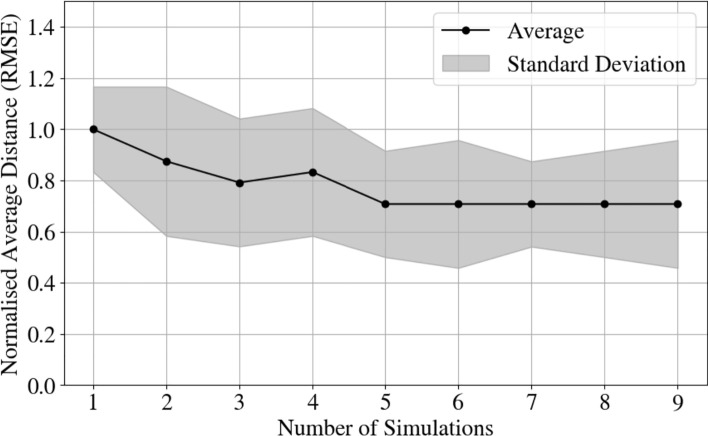
Plotting how the normalised average distance (RMSE) between the simulated and real data varies with the number of simulations

## Appendix

### Number of simulations choice

The choice of the number of simulations *N* is important to ensure robustness and validity of the output, whilst limiting computational cost. The value should be high enough to ensure the simulation reaches convergence, without unnecessary runs. This value was calculated as *N=5* to ensure stability of the output using a coarse sensitivity analysis (Fig. 4).
